# Direct α-synuclein promoter transactivation by the tumor suppressor p53

**DOI:** 10.1186/s13024-016-0079-2

**Published:** 2016-02-02

**Authors:** Eric Duplan, Cécile Giordano, Frédéric Checler, Cristine Alves da Costa

**Affiliations:** Institut de Pharmacologie Moléculaire et Cellulaire, UMR7275 CNRS/UNSA, team labeled “Fondation pour la Recherche Médicale” and “Laboratory of Excellence (LABEX) Distalz”, 660 route des Lucioles, 06560 Sophia-Antipolis, Valbonne France

**Keywords:** p53, α-synuclein, Transcription

## Abstract

**Background:**

Parkinson’s disease (PD) is a motor disease associated with the degeneration of dopaminergic neurons of the *substantia nigra pars compacta*. p53 is a major neuronal pro-apoptotic factor that is at the center of gravity of multiple physiological and pathological cascades, some of which implying several key PD-linked proteins. Since p53 is up-regulated in PD-affected brain, we have examined its ability to regulate the transcription of α-synuclein, a key protein that accumulates in PD-related Lewy bodies.

**Results:**

We show that pharmacological and genetic up-regulation of p53 expression lead to a strong increase of α-synuclein protein, promoter activity and mRNA levels. Several lines of evidence indicate that this transcriptional control is due to the DNA-binding properties of p53. Firstly, p53 DNA-binding dead mutations abolish p53 regulation of α-synuclein. Secondly, the deletion of p53 responsive element from α-synuclein promoter abrogates p53-mediated α-synuclein regulation. Thirdly, gel shift and chromatin immunoprecipitation studies indicate that p53 interacts physically with α-synuclein promoter both in vitro and in a physiological context. Furthermore, we show that the depletion of endogenous p53 in cells as well as in knockout mice down-regulates α-synuclein transcription.

**Conclusions:**

Overall, we have identified α-synuclein as a new transcriptional target of p53 and delineated a cellular mechanism feeding the accumulation of toxic aggregated α-synuclein in PD. This original α-syn regulatory mechanism may be central to PD-related cell death and may lead to novel opportunities to design alternative neuroprotective strategies in PD.

**Electronic supplementary material:**

The online version of this article (doi:10.1186/s13024-016-0079-2) contains supplementary material, which is available to authorized users.

## Background

Parkinson’s disease (PD) is a major age-related motor disease in which several causative genes have been identified. Amongst these genes, α-synuclein (α-syn) has caught a special attention given its important role in PD etiology [1, 2]. Thus, α-syn is a small phosphoprotein, that accumulates in intracellular inclusions named Lewy bodies in most of sporadic and genetic PD cases [3]. We previously showed that it exerts an anti-apoptotic phenotype by down-regulating p53 expression and transcriptional activity in neuronal cells [4, 5]. Importantly this neuroprotective phenotype can be abrogated by various processes leading to its accumulation and/or aggregation. Thus, we have shown that 6-hydroxydopamine, an endogenously produced dopamine catabolite frequently used to trigger PD *ex-vivo* and in vivo*,* leads to α-syn aggregation and consequently, abolishes α-syn ability to repress p53 [6].

It is interesting to note that several proteins such as DJ-1 and parkin, which when mutated trigger familial forms of PD, have been shown to regulate and, to be regulated by p53 [7–11]. In this context, the ability of α-syn to regulate p53 led us to postulate that p53 could also control α-syn levels as part of a feedback process driving their cellular homeostasis in neurons. Given the canonical transcription factor properties of p53, we have examined the putative p53-dependent control of α-syn transcription in a pathophysiological context. Our study demonstrates that both selective pharmacological treatments and genetic manipulation of p53 by overexpression or invalidation approaches trigger a modulation of α-syn transcription by a mechanism implying the physical interaction of p53 with α-syn promoter. Thus, the deletion of putative p53 responsive element of α-syn mouse promoter leads to full abolishment of p53-mediated α-syn transcription up-regulation. ChIP (chromatin immunoprecipitation) and gel shift analyses demonstrate that the p53 and α-syn interaction occurs in physiological context in absence of any transcription co-factor. Importantly, analysis of α-syn protein and mRNA levels in p53 knockout mouse brain further documents the transcriptional control of α-syn by p53 in vivo. Overall, our data identify α-syn as a novel p53 transcriptional target and thus, delineate a functional interplay driving their cellular homeostasis. Our study pinpoints to the fact that the disruption of this cellular dialogue linked to the dysfunction of any of these two partners may contribute to PD pathology.

## Results

### Pharmacological manipulation of p53 affects α-syn transcription

**Fig. 1:**
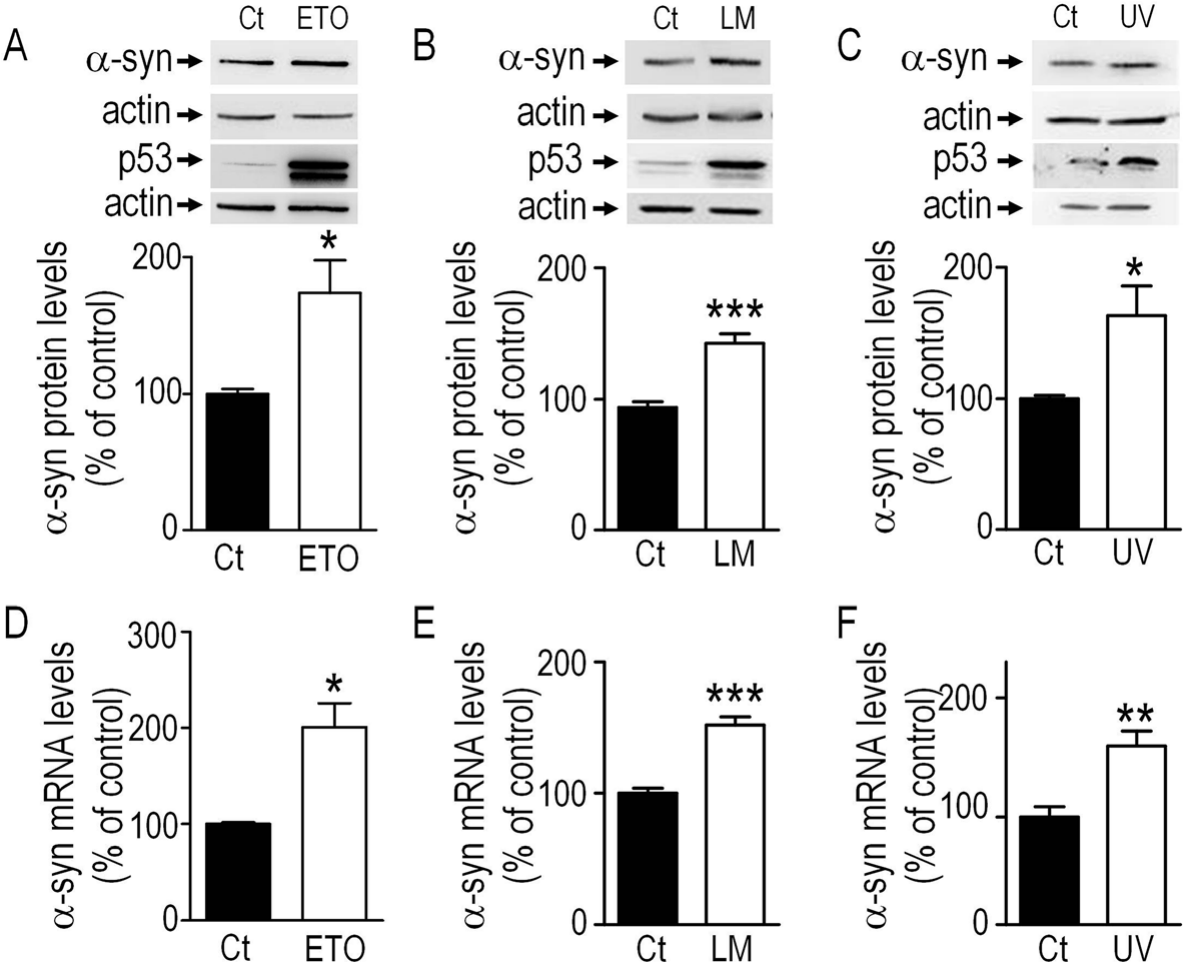
Effects of UV irradiation and pharmacological activation of p53 on α-syn protein and mRNA levels. SH-SY5Y cells were treated with vehicle (Ct) or with etoposide (ETO, 150 μM, **a** and **d**) leptomycin (LM, 10 nM, **b** and **e**) and UV irradiation (UV, **c** and **f**) then α-syn protein (**a**-**c**) and mRNA levels (**d**-**f**) were analyzed as described in the Methods section. p53 and actin immunoreactivities are provided as read-out of p53 activation and gel loading controls respectively (**a**-**c**, upper panels). Bars represent the means ± SEM of 3 independent experiments performed in duplicates and are expressed as percentage of vehicle-treated or non-irradiated control cells. Statistical analyses were performed with GraphPad Prism software (www.graphpad.com version 4.00 for Windows, San Diego, California USA) by using homoscedastic, unpaired Student’s t-test. Significant differences are: *p < 0.05, **p < 0.01, ***p < 0.001

p53-associated transcriptional function can be activated by several pharmacological compounds, either triggering post-transcriptional protein stabilization through enhanced phosphorylation (etoposide, ETO) or favoring its nuclear localization (leptomycin B, LM) where it is protected from proteasome-driven degradation [12]. p53 activity can also be activated by physical treatment such as ultra violet (UV) irradiation. We took advantage of this range of cellular challenges to examine whether p53 modulation could indeed influence α-syn mRNA and protein levels in the SH-SY5Y dopaminergic neuroblastoma cell line. As expected, all pharmacological treatment (12 hours) and physical challenges (see Methods section) trigger enhanced expression of p53 (Fig. 1a-c, upper panels). Interestingly, they concomitantly increase both α-syn protein (ETO, +95.7 % ± 29 of control untreated cells, n = 6, Fig. 1a; LM, +48.5 % ± 8.5, n = 9, Fig. 1b; UV, +63 % ± 22.8, n = 7, Fig. 1c) and mRNA (ETO, +97.8 % ± 23.3, n = 6, Fig. 1d; LM, +51.9 % ± 7.3, n = 9, Fig. 1e; UV, +59.7 % ± 15.7, n = 7, Fig. 1f) levels.

### Overexpressed and endogenous p53 control α-synuclein transcription in cells and in mouse brain

**Fig. 2:**
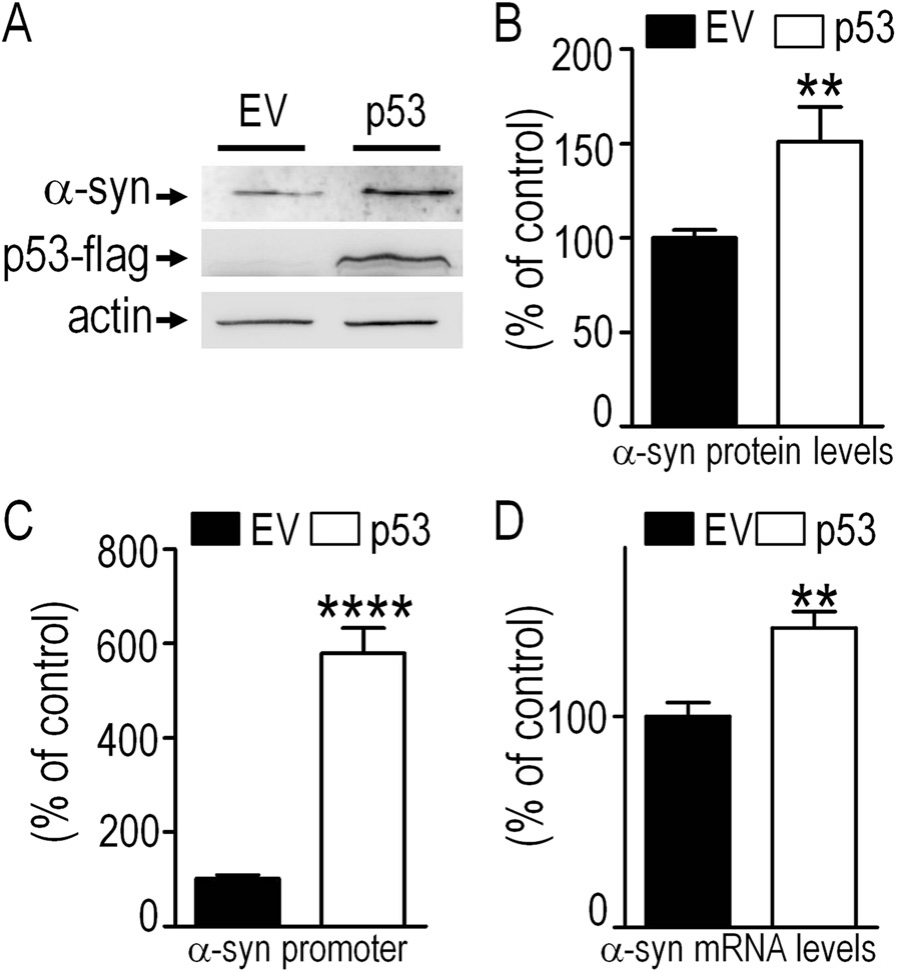
Overexpressed p53 controls α-syn transcription. SH-SY5Y cells transiently transfected with an empty vector (EV, black bars) or wild-type p53 cDNA (p53, white bars) were assessed for α-syn protein (**a** and **b**), promoter transactivation (**c**) and mRNA levels (**d**). Bars in **a**-**d** represent the means ± SEM of 3-4 independent experiments performed in duplicates or triplicates and are expressed as percentage of control EV cells. Statistical analyses were performed with GraphPad Prism software by using homoscedastic, unpaired Student’s t-test. Significant differences are: **p < 0.01, and ****p < 0.0001

**Fig. 3:**
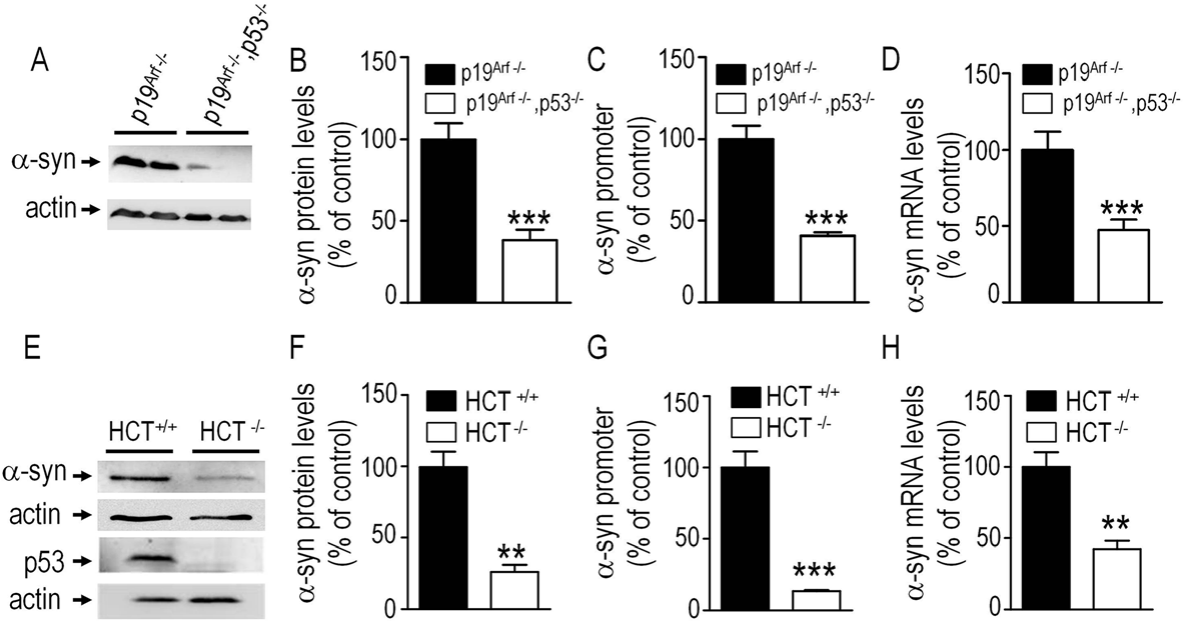
Mouse and human endogenous p53 control α-syn transcription. Control mouse fibroblasts (MEF, p19^arf-/-^, black bars) or MEF devoid of p53 (p19^arf-/-^ p53^-/-^, white bars) were assessed for α-syn protein (**a** and **b**), promoter transactivation (**c**) and mRNA levels (**d**) in basal conditions as described in the Methods section. α-syn protein (**e**, **f**), promoter transactivation (**g**) and mRNA levels (**h**) were analyzed in HCT116 control (HCT^+/+^, black bars) or *p53*-deficient (HCT^-/-^, white bars) cells as described in the Methods section. Bars in A-D represent the means ± SEM of 3-4 independent experiments performed in duplicates or triplicates and are expressed as percentage of control p19^arf-/-^ (**a-d**) or HCT^+/+^ (**e-h**) cells. Actin expression is provided as a gel loading control in (**a**, **e**). Statistical analyses were performed with GraphPad Prism software by using homoscedastic, unpaired Student’s t-test. Significant differences are: **p < 0.01, and ***p < 0.001

We have examined the influence of ectopic and endogenous p53 on α-syn protein and mRNA levels as well as on α-syn mouse promoter-linked luciferase activity (see Methods). Thus, as showed in Fig. 2 (panels a-d) 24 hours post-transfection, transiently overexpressed p53 in SH-SY5Y cells (see Fig. 2a) triggers an up-regulation of α-syn protein expression (+51.2 % ± 18.9, n = 9, Fig. 2a and b) and mouse wild-type full length α-syn promoter transactivation measured as above (+478.2 % ± 54.8, n = 12, Fig. 2c) and mRNA levels (+41.8 % ± 10.2, n = 6, Fig. 2d). We took advantage of a mouse embryonic fibroblast (MEF) cell line devoid of both p53 and p19^arf^ (p19^arf-/-^, p53^-/-^) that we compared to its control p19^arf-/-^ [13] to examine the contribution of endogenous p53. As illustrated in Fig. 3a-d, p53 knockout cells (white bars) display significant decreases of α-syn protein expression (-61.6 % ± 16.7, n = 9, Fig. 3a, b), mouse wild-type full length α-syn promoter transactivation (luciferase assay, see Methods) 24 hours post-transfection (-59.3 % ± 6.2, n = 12, Fig. 3c) and mRNA levels (-55.8 % ± 9.6, n = 6, Fig. 3d). Interestingly, UV treatment increases α-syn protein and mRNA levels in control p19^arf-/-^ cells (+42.6 % ± 10.6, n = 6, Additional file 1: Figure S1A and +53.2 % ± 19.9, n = 9, Additional file 1: Figure S1B, respectively) but not in fibroblasts lacking endogenous p53 (Additional file 1: Figure S1A,B white bars). As a matter of confirmation of an endogenous p53-dependent control of α-syn mRNA transcription in cell lines of human origin, we used the human colon carcinoma HCT116 (Fig. 3e-h) and haploid HAP1 (Additional file 2: Figure S2A-C) cells to compare the parental cell lines that exhibit wild-type p53 (HCT^+/+^, HAP^+/+^) to the ones in which *p53* gene is disrupted (HCT^-/-^, HAP^-/-^). Figure 3e-h shows that the full depletion of endogenous p53 (see lack of expression in Fig. 3e) also triggers a reduction of α-syn protein expression (-73.2 % ± 11.2, n = 6, Fig. 3e,f), mouse wild-type full length α-syn promoter transactivation measured as above 24 hours post-transfection (-86.4 % ± 6.3, n = 6, Fig. 3g) and mRNA levels (-57.4 % ± 11.7, n = 6, Fig. 3h) in HCT116 cells. Similar significant decreases of α-syn protein expression (-44.2 % ± 8.0, n = 8, Additional file 2: Figure S2A,B) and mRNA levels (-47.3 % ± 3.5, n = 6, Additional file 2: Figure S2C) were observed in HAP ^-/-^ cells.

**Fig. 4:**
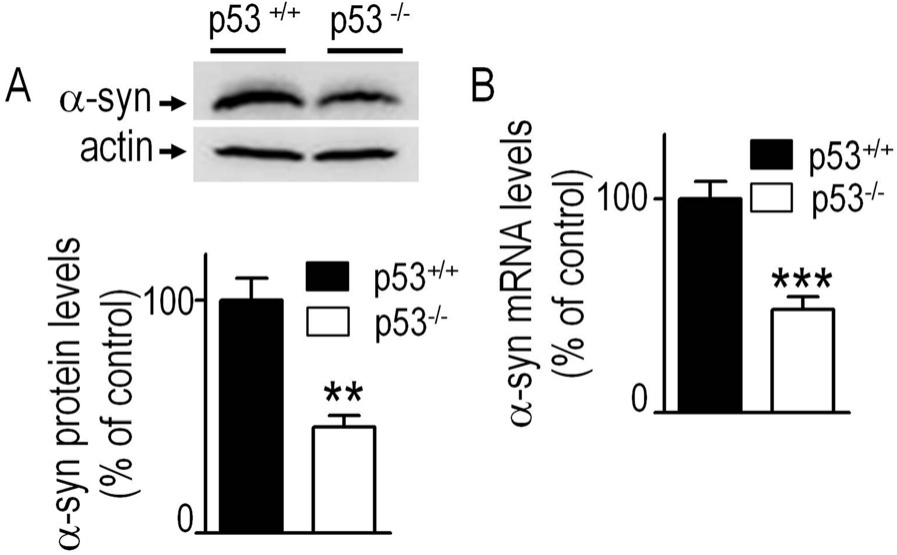
p53 control α-syn transcription in vivo. Analyses of α-syn protein (**a**) and mRNA levels (**b**) in control (p53^+/+^, black bars) or *TP53* gene invalidated (p53^-/-^, white bars) mouse brain as described in the Methods section. Bars represent the means ± SEM of n = 6 (**a**) or n = 9 animals (**b**) and are expressed as percentage of control p53^+/+^ mouse brain samples. Actin expression (**a**) is provided as protein loading control. Statistical analyses were performed with GraphPad Prism software by using homoscedastic, unpaired Student’s t-test. Significant differences are: **p < 0.01, and ***p < 0.001

In order to establish that our ex-vivo observations also stand in vivo, we have compared α-syn protein (left brain hemisphere) and mRNA status (right brain hemisphere) of wild-type mouse harboring (p53^+/+^) or lacking (p53^-/-^) endogenous p53. p53 knockout mouse brains show a similar reduction of α-syn protein expression (-58.9 % ± 11.6, n = 6, Fig. 4a) and mRNA levels (-51.8 % ± 10.1, n = 9, Fig. 4b). Overall, the above-described data demonstrate that pharmacological modulation or genetic manipulation of endogenous p53 control α-syn mRNA transcription and protein levels in various cell types from both murine and human origin as well as in mouse brain.

### p53 DNA-binding dead-mutations abolish α-synuclein transcriptional regulation *ex-vivo*

**Fig. 5:**
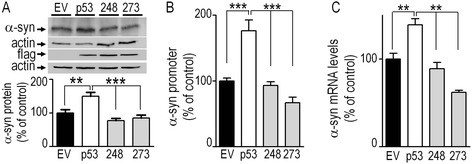
Effect of mutated p53-mediated α-syn regulation ex-vivo. α-Synuclein protein (**a**), promoter transactivation (**b**) and mRNA levels (**c**) were analyzed after transient overexpression of empty pcDNA3.1 vector (EV, black bars) or vectors encoding for wild-type p53 (p53, white bars), R248W or R273H mutants of p53 (248 and 273, gray bars) in SH-SY5Y cells as described in the Methods section. Representative gels of p53 (flag) and actin immunoreactivities (**a**) are provided to illustrate transfection efficiency and control of gel loading, respectively. Bars represent the means ± SEM of 3 independent experiments performed in duplicates (**a**, **c**) or triplicates (**b**) and are expressed as percent of EV- transfected cells. Statistical analyses were performed with GraphPad Prism software by using One-way ANOVA analysis of variance coupled to a Newman Keuls post-hoc test. Significant differences are: **p < 0.01, and ***p < 0.001

In order to establish if the transcriptional regulation of α-syn was associated to the DNA-binding properties of p53 underlying its transcription factor activity, we have analyzed the impact of two p53 mutations (R273H and R248W) known to disrupt this function. These extremely frequent (hot spot) cancer-related p53 mutations were selected due their extensive characterization regarding their impact to p53 DNA-binding properties [14, 15]. As expected, Fig. 5 first confirms that the overexpression of wild-type p53 in SH-SY5Y cells (see expression in Fig. 5a, upper panel) increases α-syn protein expression (+49.8 % ± 15.3, n = 12, Fig. 5a), mouse wild-type full length α-syn promoter transactivation measured by means of the luciferase reporter gene activity assay (+76.1 % ± 17.1, n = 9, Fig. 5b) and mRNA levels (+39 % ± 9.6, n = 6, Fig. 5c). However, while transient cDNA transfections of wild-type and mutated p53 led to very similar p53 expressions (Fig. 5a, upper panel), both mutations impair p53 ability to up-regulate α-syn protein expression (Fig. 5a), promoter transactivation (Fig. 5b) and mRNA levels (Fig. 5c).

### α-syn is a direct p53 transcriptional target

**Fig. 6:**
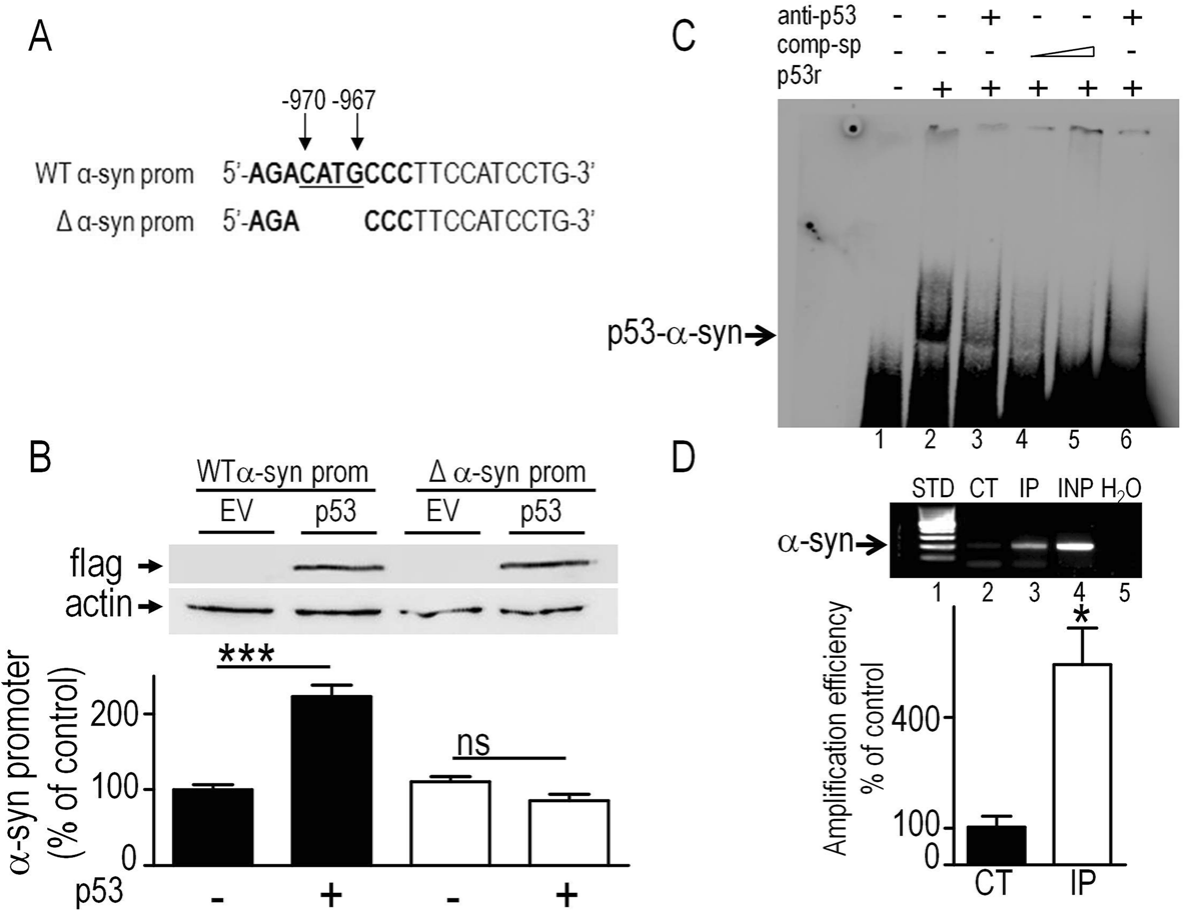
p53 interacts physically with murine α-syn promoter. **a** Scheme of the region of wild-type α-synuclein promoter (WT α-syn prom) with the p53 DNA-binding motif indicated in bold. The -970-967 (5’-CATG-3’) region underlined has been deleted (Δα-syn prom). **b** SH-SY5Y cells were co-transfected with either empty pcDNA3 vector (EV, lanes -) or p53 cDNA (p53, lanes +) and WT α-syn prom (black bars) or Δα-syn prom (empty bars) constructs then promoter activities were measured as described in the Methods section. p53 expression was controlled by anti-Flag antibodies as described in Methods. Bars represent the means ± SEM of 4 independent experiments performed in triplicates and are expressed as percentage of control EV-transfected cells. **c** EMSA analysis of the interaction of wild-type recombinant p53 (p53r) with α-syn-derived biotinylated probe encompassing the consensus sequence shown in (**a**). Reactions were carried out in absence (-) or in the presence (+) of either an excess of specific cold probes (comp-sp, see lanes 4, 5) or p53-directed antibodies (anti-p53 pab421/DO1, lanes 3, 6) and analyzed as described in the Methods. Free probe control is shown in lane 1. **d** ChIP analysis of the interaction between endogenous p53 with α-syn endogenous promoter in MEF cells by endpoint semi-quantitative PCR (upper gel) or real time PCR (histogram) as described in Methods. In (**d**), lanes 1–5 correspond to 100 bp DNA ladder (STD), normal mouse IgG ChIP (CT), p53 ChIP (IP), input (INP) and no template control (H_2_O), respectively. Statistical analysis was performed with GraphPad Prism software by using either One-way ANOVA analysis of variance coupled to a Newman Keuls post-hoc test (**b**) or homoscedastic, unpaired Student’s t-test (**d**). Significant differences are: *p < 0.05, ***p < 0.001, and ns for non-significant

The question arose as to whether p53 could directly or indirectly modulate α-syn promoter transactivation. An *in silico* bioinformatic search for p53 DNA-binding consensus motif [16] identified a unique motif corresponding to half of the canonical p53 responsive element in the mouse α-syn promoter (-970 → -967, Fig. 6a). We first examined the influence of the deletion of this putative p53 responsive element on p53-mediated modulation of α-syn transcription. First, as expected, we confirmed that, conversely to the phenotype observed after depletion of endogenous p53, its overexpression (see Fig. 6b, upper panel) increases α-syn wild-type full length α-syn mouse promoter transactivation (+123.1 % ± 16.6, n = 12, Fig. 6b compare black bars). This p53-related phenotype is completely abolished by the removal of the p53-binding domain on the α-syn promoter construct (Δ α-syn prom., Fig. 6b, compare white bars). Two lines of additional data definitely flag-up α-syn as a transcriptional target of p53. Firstly, we performed electrophoretic mobility shift assay (EMSA) where recombinant wild-type p53 protein and biotinylated α-syn-derived probe encompassing its p53 DNA-binding motif were incubated together. This bi-molecular reaction allows monitoring a genuine physical interaction between the two partners in absence of any cellular modulator or intermediate. Figure 6c illustrates the gel shift elicited by p53/α-syn probe physical interaction (Fig. 6c, compare lanes 1 and 2). The specificity of this interaction was demonstrated by its blockade by pre-incubation of p53 recombinant protein with two distinct p53 antibodies pab421/DO1 (anti-p53, lane 3 and 6 respectively) or by incubation with an excess of specific non-biotinylated α-syn probes (comp-sp, lanes 4 and 5). Secondly, we showed by ChIP experiments that endogenous p53 immunoprecipitation yielded a α-syn promoter PCR-amplified fragment containing the p53 DNA-binding motif (Fig. 6d, compare control lane 2 with IPed lane 3). This set of deletion-based analysis, gel shift data and ChIP experiments all support our *in silico* prediction and demonstrate that α-synuclein behaves as a genuine direct p53 transcriptional target.

## Discussion

p53 is a transcription factor, which main physiological function is to regulate genes involved in the control of cell cycle, DNA repair and apoptosis [17, 18]. Thus, p53-associated gene modulation normally stops cell growth by blocking the cell cycle at the G1/S phase and triggers a pro-apoptotic cascade [18]. Moreover, p53 is a pro-apoptotic protein strongly linked to several neurodegenerative disorders, amongst which Parkinson’s disease [19]. Thus, *post-mortem* studies of PD-affected patients have shown abnormal p53 expression and the evaluation of the impact of toxin-induced PD demonstrated a strong contribution of p53 in dopaminergic cell death. Finally, several PD-causative gene products including DJ-1 and parkin, have been shown to control and to be controlled by p53 *ex-vivo* and in vivo [19, 20].

Our study unravels for the first time α-syn as an additional transcriptional target of p53. This conclusion stands on five independent lines of data. Firstly, overexpression of p53 or its pharmacological activation enhance α-syn promoter activity, mRNA and protein levels in human and murine cells from various origins while *p53 gene* depletion triggers the opposite phenotype in cells as well as in vivo, in mouse brain; secondly, DNA-binding dead mutations of p53 abolish p53-induced α-syn transactivation both *ex-vivo*; thirdly, the deletion of a putative p53 DNA-binding element on the α-syn promoter prevents p53-mediated increase of α-syn promoter activation; fourth, EMSA experiments indicated that recombinant p53 and α-syn promoter-derived probes physically interact directly without the need of any cofactor; fifthly, ChIP analysis demonstrates the physical association of endogenous p53 with α-syn promoter in an *ex-vivo* physiological context.

Very few works aimed at studying the transcriptional regulation of α-syn have been performed. Importantly, previous data indicated that α-syn mRNA levels could be modulated by PD inducers like MPTP (1-Methyl-4-phenyl-1,2,3,6-tetrahydropyridine hydrochloride) [21] and appeared increased in PD-affected brains [22]. Thus, our data allowed the establishment of a molecular mechanism that ultimately may contribute to the transcriptional modulation of α-syn in sporadic PD.

We previously established that α-syn lowers both p53 protein levels and transcriptional activity in neuronal cell lines [5] and that this control was abolished by 6-hydroxydopamine that triggers both α-syn oxidation and aggregation and proteasomal inhibition [6]. This study, together with our present data, unravels a functional interplay between α-syn and p53 that could be disrupted in pathological conditions. Thus, in normal conditions, α-syn represses p53 [1, 5]. This reduction of p53 triggers lowering of α-synuclein transactivation and protein levels (present study), which in turn restores p53 physiological levels. We speculate that this reciprocal physiological control, which allows the homeostasis of both proteins, may be impaired in PD due to dysfunctions of either α-syn or p53. Thus, in those cases where the function of α-syn is hampered by its aggregation or by pathogenic mutations, one can anticipate the accumulation of p53 and by consequence the establishment of a deleterious p53-mediated transcriptional feeding of α-syn increase. Alternatively, since p53 is at the crossroad of multiple signaling cascades involved in PD pathogenesis, one can suppose that its abnormal activation could lead to excessive α-syn transcription and production. Since α-syn aggregation is strongly linked to its protein levels, one can envision that p53 could contribute to α-syn aggregation and toxicity via exacerbation of its transcriptional regulation.

## Conclusions

Our work demonstrates that α-syn promoter harbors a functional p53 responsive element. Thus, p53 interacts with the mouse α-syn promoter and up-regulates α-syn transcription. Pharmacological and genetic manipulation of p53 impacts α-syn regulation and importantly, endogenous p53 regulates α-syn transcription *ex-vivo* and in vivo. This first delineation of α-syn as a physiological transcriptional target of p53 unraveling a functional dialogue between these two proteins allows proposing that, in a PD-linked pathological context, α-syn toxicity could be likely the consequence of a loss of its physiological interplay with p53.

## Methods

### Cellular and animal models

Mouse Embryonic Fibroblasts (MEF), Human Colorectal adenocarcinoma (HCT116), SH-SY5Y human neuroblastoma, cell lines were cultured in Dulbecco’s modified Eagle’s medium (DMEM) supplemented with 10 % fetal calf serum, penicillin (100 U/ml) and streptomycin (50 μg/ml) and incubated at 37 °C in a 5 % CO2 atmosphere. HAP1 cells purchased from Horizon Genomics (HAP1 p53^+/+^) is a haploid human cell line that was derived from KBM-7 cells (see [23] HAP1 clone 1068-1 (HAP1 p53^-/-^) was engineered using CRISPR/cas9 and contains one base insertion in exon 6 leading to a frame-shift in TP53 coding sequence. HAP1 cells were cultured in Iscove’s Modified Dulbecco’s Medium (IMDM) with 10 % fetal calf serum, penicillin (100 U/ml) and streptomycin (50 μg/ml). Immortalized mouse embryonic fibroblasts invalidated for *p53* or for *p53* and *p19*^*arf*^ genes were kindly provided by Drs. M. Roussel (St. Jude Children’s Research Hospital Memphis, TN, USA) whereas the cell lines HCT116 invalidated or not for *p53* were provided by J.C. Bourdon (University of Dundee, Dundee, UK). *p53* knockout mice have been provided by Dr. M. Serrano (Spanish National Cancer Research Center, Madrid, Spain).

### Pharmacological and UV irradiation-mediated modulation of p53

Activation of p53 was achieved by UV-light irradiation of the cells by means of a cross-linker apparatus (254 nm bulb). Various cell types were cultivated in 35 mm dishes and submitted to a double optimal crosslink treatment (60 sec, 120 mJ/cm^2^) when they reached 80 % confluence then left 24 hours at 37 °C in a 5 % CO_2_ atmosphere. The pharmacological modulation of p53 was obtained after 12 hours incubations with etoposide (150 μM) or leptomycin (10 nM). After treatments, cells were recovered and protein and RNA analyses were monitored as described below.

### Western-blot protein analysis

α-syn, p53 and actin expressions were analyzed in various cell lines and mouse brain homogenates (50-200 μg of proteins) then loaded on 12-16 % sodium dodecyl sulphate polyacrylamide gel electrophoresis (SDS-PAGE) gels and wet-transferred to Hybond-C (Amersham Life Science) membranes. Transferred proteins were immunoblotted using anti-α-syn (#2642, Cell Signaling), anti-flag (clone M2, F3165, Sigma), anti-p53 (CM1, provided by J.C. Bourdon) and anti-actin (clone AC-74, A5316, Sigma) antibodies. Immunological complexes were revealed with either anti-rabbit or anti-mouse IgG-coupled peroxidase antibodies (Jackson ImmunoResearch) by the electrochemiluminescence detection method (Roche Diagnostics S.A.S). Chemiluminescence was recorded using a luminescence image analyzer LAS-4000 (Raytest, Fuji) and quantifications of non-saturated images were performed with the FUJI Film Multi Gauge image analyzer software.

### Plasmid constructs and transfections approaches

The generation of wild-type and R273H flag-tagged p53 mutant of human p53 flag-tagged coding sequences in the mammalian expression vector pcDNA3.1 (+) has been extensively described [24]. To generate the R248W p53 mutant, we used the site‐directed mutagenesis kit from Stratagene along with the forward p53R248WS (5’-GGG-CGG-CAT-GAA-CTG-GAG-GCC-CAT-CCT-CAC-C-3’) and the reverse p53R248WAS (5’-GGT-GAG-GAT-GGG-CCT-CCA-GTT-CAT-GCC-GCC-C-3’) primers.

To clone the mouse *SNCA* promoter, 200 ng of mouse fibroblast genomic DNA, obtained using the kit QIAamp DNA mini kit (Qiagen) were added to a high fidelity PCR reaction mix, with forward primer 5’-CGA-CGC-GTG-GAG-GAG-CTT-GGC-ACT-CAA-ATC-3’ containing a MluI restriction site and the reverse primer 5′-GAA-GAT-CTG-GCT-AAA-GAT-GTA-TTT-TTG-CTC-CAC-ACT-AG-3′ containing a BglII restriction site. The 2251 nucleotides amplicon (DNA fragment immediately upstream the translation initiation codon of mouse *SNCA* gene) has been cloned between the MluI and BglII sites of the pGL3 basic vector (containing the sequence coding for luciferase) from Promega. This pGL3 vector containing the mouse *SNCA* promoter served as a template to generate the promoter deleted of the 5’-CATG-3’ nucleotides that constitute part of the p53 putative binding site. The primers used were 5′-CTT-TCC-TTT-CGC-TGG-AGA-CCC-TTC-CAT-CCT-GTC-3′ (forward primer) and 5′-GAC-AGG-ATG-GAA-GGG-TCT-CCA-GCG-AAA-GGA-AAG-3′ (reverse primer). All the constructs were verified by full sequencing. Transient transfections of SH-SY5Y and HAP1 cells were carried out by means of lipofectamine 2000 (Invitrogen) according to the manufacturer’s instructions.

### Luciferase-based reporter assays

The transactivation of the wild-type and mutated *SNCA* mouse promoter described in the plasmid constructs section was followed by recording the luciferase reporter gene activity 24 hours after co-transfection of 0,5–1 μg of the above cDNAs and 0,2–0,5 μg of β-galactosidase cDNA (in order to normalize for transfection efficiencies) by means of lipofectamine 2000 (Invitrogen) according to the manufacturer’s instructions (Invitrogen). When necessary, in a subset of experiments, 0,5–1 μg of empty pcDNA3.1, wild type or mutated p53 were co-transfected.

### RNA extraction, reverse transcription and real-time PCR analysis

RNA from cells and RNA later (Qiagen) stabilized mouse brains (right hemisphere) were extracted and treated with DNAse using RNeasy or RNeasy Plus Universal Mini kits respectively following manufacturer’s instructions (Qiagen). Two μg of total RNA were reverse transcribed (GoScript Reverse Transcriptase, Promega) using Oligo-dT priming. Then, samples were subjected to real-time PCR by means of a Rotor-Gene 6000 apparatus (Qiagen), using the SYBR Green detection protocol. Gene-specific primers were designed with the Universal Probe Library Assay Design Center software (Roche Applied Science). Expression levels of mouse (forward: 5’-TGG-CAG-TGA-GGC-TTA-TGA-AA-3’; reverse: 5’-GCT-TCA-GGC-TCA-TAG-TCT-TGG-3’) and human (forward: 5’-GCC-TTT-CCA-CCC-TCG-TGA-G-3’; reverse: 5’-ACT-GTC-GTC-GAA-TGG-CCA-C-3’) α-syn amplification products were normalized for RNA concentrations by mouse γ-actin (forward: 5′-CAC-CAT-CGG-TTG-TTA-GTT-GCC-3′; reverse: 5′-CAG-GTG-TCG-ATG-CAA-ACG-TT-3′) or human GAPDH (forward: 5′-TGG-GCT-ACA-CTG-AGC-ACC-AG-3′; reverse: 5′-CAG-CGT-CAA-AGG-TG G-AGG-AG-3′) mRNA expression levels.

### Electrophoretic mobility gel shift assay (EMSA)

EMSA was performed by means of a commercial Gel shift Chemiluminescent EMSA Assay Kit provided by Active Motif. In brief, purified wild-type p53 recombinant protein (200 ng; SC4246, Santa Cruz) was incubated in 1X binding buffer (Promega) containing 100 ng poly (dI-dC), dithiothreitol (10 mM) and Bovine Serum Albumin (1 mg/ml) at 4 °C for 30 min with 20 fmol of double stranded 5’ biotin end-labeled *SNCA*-derived oligonucleotides (forward: 5′-CCT-TTC-GCT-GGA-GAC-ATG-CCC-TTC-CAT-CCT-GTC-AAA-GCC-C-3′; reverse: 5′-GGG-CTT-TGA-CAG-GAT-GGA-AGG-GCA-TGT-CTC-CAG-CGA-AAG-G-3′) encompassing the putative p53 responsive element. Protein-probe complexes were then resolved by electrophoresis on a 5 % native polyacrylamide gel at 4 °C, transferred to a positively charged nylon membrane (Thermo Scientific), cross-linked (UV-light cross-linker equipped with a 254 nm bulb) and revealed by means of streptavidin conjugated to horseradish peroxidase (HRP) and a chemiluminescent substrate. When indicated, in order to confirm specific DNA-protein interaction, we performed a 15–30 min pre-incubation at 4 °C with either an excess (4 pmol) of unlabeled specific and non-related control DNA or pab421 before adding the biotin-labeled probes.

### Chromatin Immunoprecipitation assay (ChIP)

ChIP assay was performed according to EZ- ChIP kit instructions (Millipore). Briefly, 10^7^ cells were fixed with formaldehyde (1 % final concentration), treated with glycine to quench unreacted formaldehyde and recovered in cold phosphate buffered saline (PBS) containing the protease inhibitor cocktail II. Pelleted cells were lysed in the SDS lysing buffer and sonicated on ice in order to obtain chromatin fragments of about 200-500 bp in size. After a preclearing step using protein G Agarose, immunoprecipitation was performed either with anti-p53 primary antibody (pab421, Enzo Life Sciences) or normal mouse IgG as a negative control. Immunocomplexes were then incubated with a solution of protein G-agarose. After elution of the immunocomplexes from beads, crosslinks were reversed and RNA and protein eliminated by RNAse and proteinase K treatments. DNA was purified and subjected to a standard end-point and real-time PCR using primers (forward: 5′-CGC-CTA-GAG-AAG-ACC-AAC-TAC-AGC-TGC-3′; reverse: 5′-GCA-CTA-AGC-TTC-CAC-CAT-CCA-GCA-CTC-AAC-3′) specific for the -1061/-852 DNA region (210 bases-long amplicon) upstream the start codon of mouse *SNCA* gene. We calculated the enrichment as the ratio of the amplification efficiency of the ChIP sample over that of the IgG.

### Statistical analysis

Statistical analyses were performed with GraphPad Prism software (www.graphpad.com version 4.00 for Windows, San Diego, California USA). All groups of samples analyzed by Student’s t-test have passed a normality test to assure Gaussian distribution of values and precision concerning the type of test (unpaired versus paired and homoscedastic versus heteroscedastic) are provides in figures legends. Analysis of more than two groups of variables (normality test passed) was performed by One-way ANOVA with Newman-Keuls’s post-hoc test. Significant differences are: *p < 0.05, **p < 0.01, ***p < 0.001, ****p < 0.0001 and ns = non significant.

## Additional files

Additional file 1:
**UV-induced increase of a-syn expression is abolished by** ***TP53*** **invalidation in MEF cells.** Control (MEF, p19^arf-/-^, black bars) or p53-deficient (p19^arf-/-,^ p53^-/-^, white bars) mouse fibroblasts were assessed for α-syn protein (**A**) and mRNA levels (**B**) in basal conditions (Ct) or after UV-treatment (UV) as described in the Methods section. Bars represent the means ± SEM of 3-4 independent experiments performed in triplicates (**A**) or duplicates (**B**) and are expressed as percentage of control p19^arf-/-^ cells. Actin expression (**A**) is provided as protein loading control. Statistical analyses were performed with GraphPad Prism software by using One-way ANOVA analysis of variance coupled to a Newman Keuls post-hoc test. Significant differences are: *p < 0.05, ***p < 0.001, and ns for non-significant. (JPG 196 kb) Additional file 2:
**Influence of endogenous human** ***TP53*** **invalidation on α-syn regulation in human cells.** α-syn protein (**A**, **B**) and mRNA levels (**C**) were analyzed in HAP1 control (HAP^+/+^, black bars) or *p53*-deficient (HAP^-/-^, white bars) cells as described in the Methods section. Bars represent the means ± SEM of 3 independent experiments performed in duplicates and are expressed as percent of control HAP1 (HAP^+/+^) cells. Actin expression is provided as a gel loading control in (**A**). Statistical analyses were performed with GraphPad Prism software (www.graphpad.com version 4.00 for Windows, San Diego, California USA) by using homoscedastic, unpaired Student’s t-test. Significant differences are: ***p < 0.001. (JPG 161 kb)

## Abbreviations

ChIP: chromatin immunoprecipitation assay
DMEM: Dulbecco’s modified Eagle’s medium
EMSA: electrophoretic mobility gel shift assay
ETO: etoposide
HCT116: human colorectal adenocarcinoma 116
IMDM: iscove’s modified dulbecco’s medium
LM: leptomycin
MEF: mouse embryonic fibroblasts
MPTP: 1-Methyl-4-phenyl-1,2,3,6-tetrahydropyridine hydrochloride
PD: Parkinson's disease
SDS-PAGE: sodium dodecyl sulphate polyacrylamide gel electrophoresis
UV: ultra-violet
α-syn: α-synuclein
