# Supplementary figures and images for: Direct α-synuclein promoter transactivation by the tumor suppressor p53
Source: Mol Neurodegener. 2016 Feb 2;11:13. doi: 10.1186/s13024-016-0079-2 (PMC4736712; doi:10.1186/s13024-016-0079-2)

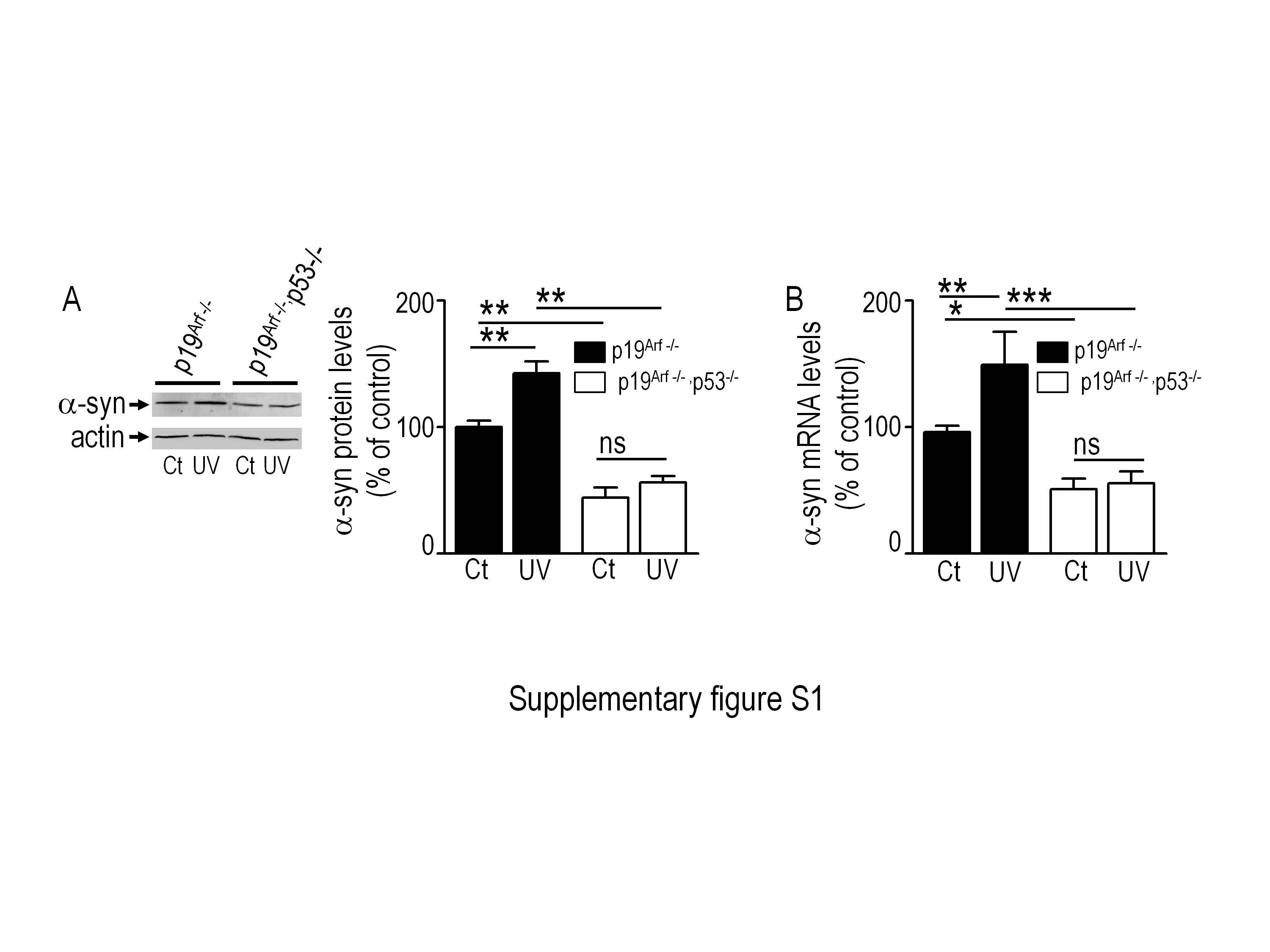

Supplement: Additional file 1: — UV-induced increase of a-syn expression is abolished by TP53 invalidation in MEF cells. Control (MEF, p19arf-/-, black bars) or p53-deficient (p19arf-/-, p53-/-, white bars) mouse fibroblasts were assessed for α-syn protein (A) and mRNA levels (B) in basal conditions (Ct) or after UV-treatment (UV) as described in the Methods section. Bars represent the means ± SEM of 3-4 independent experiments performed in triplicates (A) or duplicates (B) and are expressed as percentage of control p19arf-/- cells. Actin expression (A) is provided as protein loading control. Statistical analyses were performed with GraphPad Prism software by using One-way ANOVA analysis of variance coupled to a Newman Keuls post-hoc test. Significant differences are: *p < 0.05, ***p < 0.001, and ns for non-significant. (JPG 196 kb) [file 13024_2016_79_MOESM1_ESM.jpg]

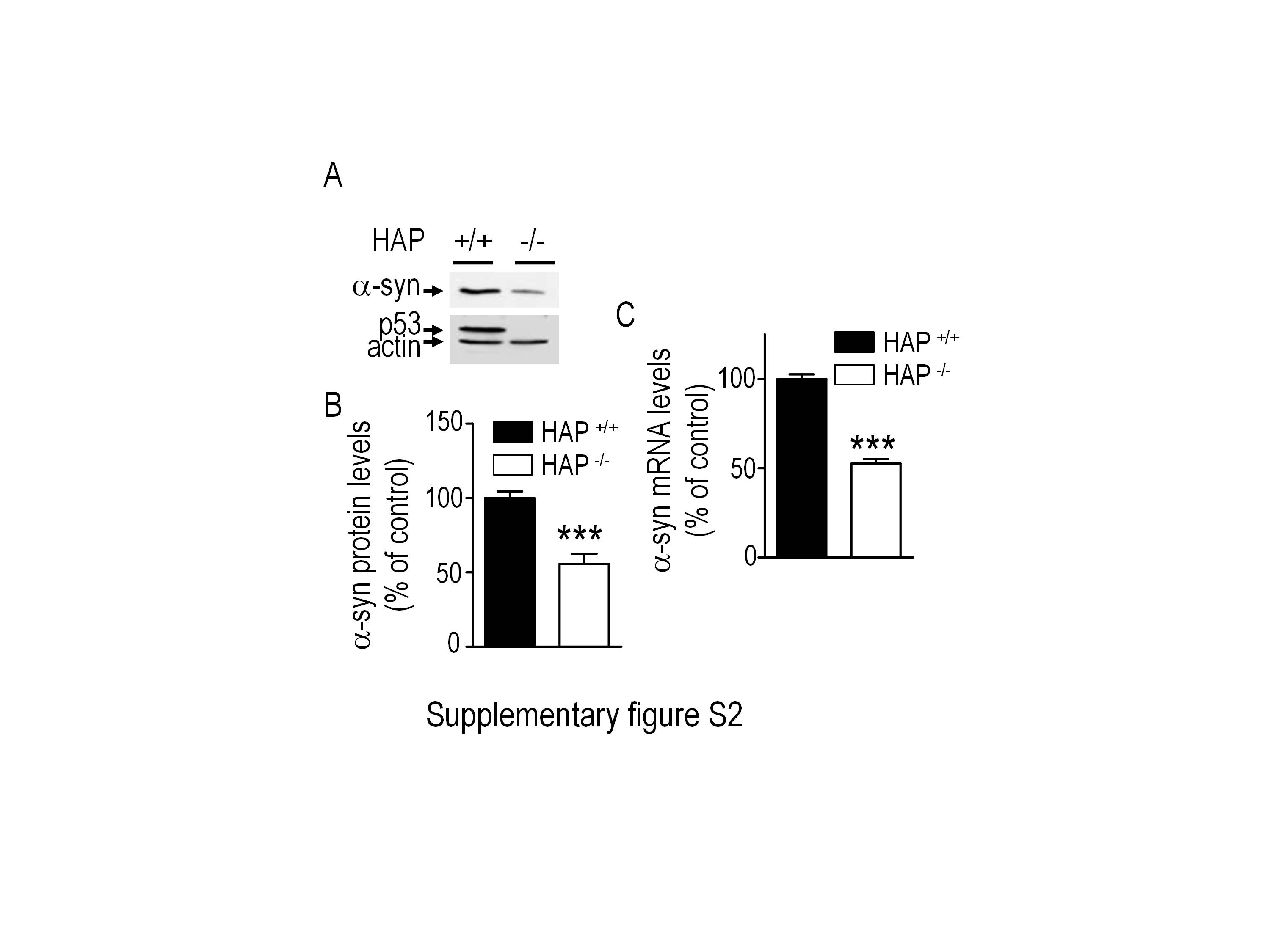

Supplement: Additional file 2: — Influence of endogenous human TP53 invalidation on α-syn regulation in human cells. α-syn protein (A, B) and mRNA levels (C) were analyzed in HAP1 control (HAP+/+, black bars) or p53-deficient (HAP-/-, white bars) cells as described in the Methods section. Bars represent the means ± SEM of 3 independent experiments performed in duplicates and are expressed as percent of control HAP1 (HAP+/+) cells. Actin expression is provided as a gel loading control in (A). Statistical analyses were performed with GraphPad Prism software (www.graphpad.com version 4.00 for Windows, San Diego, California USA) by using homoscedastic, unpaired Student’s t-test. Significant differences are: ***p < 0.001. (JPG 161 kb) [file 13024_2016_79_MOESM2_ESM.jpg]
